# Expert consensus on communicating tau PET results to persons living with MCI or dementia: Findings from a modified Delphi study

**DOI:** 10.1002/alz.71321

**Published:** 2026-04-08

**Authors:** Claire Erickson, Kyra S. O'Brien, Emily A. Largent, Kristin Harkins, Cameron Coykendall, Melanie Kleid, Justin T. Clapp, Jason Karlawish

**Affiliations:** ^1^ Banner Alzheimer's Institute Phoenix Arizona USA; ^2^ Department of Neurology University of Pennsylvania Perelman School of Medicine Philadelphia Pennsylvania USA; ^3^ Department of Medical Ethics and Health Policy University of Pennsylvania Perelman School of Medicine Philadelphia Pennsylvania USA; ^4^ Division of Geriatrics, Department of Medicine University of Pennsylvania Perelman School of Medicine Philadelphia Pennsylvania USA

**Keywords:** expert consensus, patient communication, tau positron emission tomography

## Abstract

**INTRODUCTION:**

There is interest in incorporating tau imaging into clinical care because it provides unique diagnostic and prognostic information. Yet, clinicians lack guidance on communicating results.

**METHODS:**

We conducted a modified Delphi process with practicing US‐based expert clinicians in human tau imaging. Expert clinicians were interviewed to elicit input on best practices for communicating tau positron emission tomography (PET) results to cognitively impaired patients. These data were used to develop candidate practices and statements, which expert clinicians rated across two online survey rounds.

**RESULTS:**

Eighteen expert clinicians completed interviews and both surveys. They reached consensus on 12 practices – like showing individuals their tau PET scan images – and eight statements for communicating tau PET results – one for tau alone, three for concordant amyloid and tau results, and four for discordant amyloid and tau results – to cognitively impaired patients.

**DISCUSSION:**

This study provides novel consensus recommendations for communicating tau PET results to cognitively impaired patients.

## Highlights

- Expert consensus on steps to return tau PET to patients.
- Expert consensus on language to communicate tau PET results to patients.
- The consensus‐derived practices and statements can be used to train clinicians.

## 1 BACKGROUND

Measures of beta‐amyloid have emerged as important clinical tools for persons living with mild cognitive impairment (MCI) or dementia. Amyloid positron emission tomography (PET), for example, is used to determine whether Alzheimer's disease (AD) is the likely cause of patients’ cognitive impairment, whether they are eligible for treatment with amyloid‐targeting therapies, and, for one such therapy, whether and when treatment should be stopped.^1^ As a result, substantial progress has been made toward developing guidelines and a clinical practice around communicating amyloid PET results to cognitively impaired patients.^2^, ^3^, ^4^

No comparable guidance or practice exists for communicating tau PET results to patients. The label for the one tau PET tracer currently approved by the US Food and Drug Administration (FDA), flortaucipir (Tauvid), classifies a scan as “positive” only when neurofibrillary tangles are distributed throughout the neocortex.^5^ It contains the following statement:

“A positive scan shows increased neocortical activity in posterolateral temporal, occipital, or parietal/precuneus region(s), with or without frontal activity. Neocortical activity in either hemisphere can contribute to identification of the positive pattern. A positive scan supports the presence of widely distributed tau neuropathology.”^5^

This broad and binary language is a potential shortcoming of the FDA‐approved label given growing consensus among AD experts that tau PET provides unique information and a corresponding desire to develop quantitative thresholds and staging methods.^6^, ^7^ For instance, revised criteria for the diagnosis and staging of AD, published in 2024, classify biofluid tau and tau PET as key prognostic AD biomarkers.^8^ These criteria highlight the relevance of the regional distribution of tau PET for staging disease and suggest that tau PET signal in the medial temporal region indicates early disease, moderate neocortical uptake indicates intermediate disease, and high neocortical uptake indicates advanced disease. The discordance between the FDA label and practice is gaining importance as tau PET results may soon be relevant to clinical decision‐making.^9^ Ongoing clinical trials of amyloid‐targeting therapies utilize tau PET as a secondary outcome measure,^10^ and clinical trials of tau‐targeting therapies are under way.^11^, ^12^ Beyond amyloid and tau, other markers, such as neurofilament light chain and glial fibrillary acidic protein, are also being studied for their utility in disease staging, prognosis, and treatment effects.^8^

To address the need for guidance on how to communicate tau PET results to cognitively impaired patients (and, as appropriate, to research subjects) in a more nuanced manner, we conducted a modified Delphi process with practicing clinicians who also have expertise in human tau PET imaging. Here, we report on practices and statements that achieved consensus for communication of tau PET results to individuals living with MCI or dementia.

## 2 METHODS

We interviewed US‐based, practicing clinicians with expertise in human tau PET imaging about how they communicate tau PET results to cognitively impaired patients. These qualitative data were used to develop a list of candidate practices and statements for returning tau PET results to patients. We then used a modified Delphi process to select the most acceptable and important practices and statements for the return of tau PET results.^13^ The Delphi method is useful for obtaining reliable expert consensus when empirical evidence is not available.^13^ We adhered to the Standards for Reporting Qualitative Research.^14^

The study protocol was reviewed by the University of Pennsylvania Institutional Review Board and deemed exempt. Participants reviewed a written description of the research, provided consent to participate, and were compensated up to US$200.

**RESEARCH IN CONTEXT**

**Systematic review**: To identify potential participants, the authors used the knowledge resource nomination method, a process informed by a strategic literature search using PubMed to create a list of relevant experts. To be eligible, individuals had to (1) have expertise in human tau imaging, (2) see patients clinically, and (3) reside in the United States.

**Interpretation**: Expert clinicians reached consensus on 12 practices for returning tau PET results to patients – such as showing patients their PET scan images and describing tau PET's limitations – as well as eight statements for explaining results: one when returning tau PET results alone, three for concordant amyloid and tau PET results, and four for discordant amyloid and tau PET results.

**Future directions**: Our findings begin to fill the current gap in guidance for communicating tau PET results to cognitively impaired patients. As new tracers and treatments become available, this guidance may evolve, or new guidance may be needed.

### 2.1 Expert identification

To identify potential participants, we used the knowledge resource nomination method,^15^ a process informed by a strategic literature search to create a list of relevant experts. To be eligible, individuals had to (1) have expertise in human tau imaging, (2) see patients clinically, and (3) reside in the United States.

For criterion 1, we defined an expert as an individual with a 3‐year or longer record of influential publications in human tau PET imaging, with focus on lead or senior author status. We conducted Medline searches for recent and relevant publications using combinations of keywords “Alzheimer disease” AND “positron emission tomography” AND “tau proteins.” All searches were limited to publications for the decade from 2013 to 2023. For criterion 2, we defined clinicians as individuals with clinical training who are currently evaluating – or who have within the past year evaluated – patients for cognitive impairment. Individuals’ institutional profiles were reviewed to determine whether they had clinical training. For criterion 3, we limited enrollment to US‐based individuals, as reflected in their institutional profile, to ensure an understanding of the US healthcare system. Contact information was pulled from publications (e.g., for corresponding authors), institutional websites, or personal contacts. We produced an initial list of 55 individuals meeting eligibility criteria.

### 2.2 Sampling and recruitment

While the optimal sample size for Delphi studies is debated, our goal was to enroll a minimum of 10 expert clinicians or “panelists.” Larger samples are thought to produce more stable results (e.g., consistency in the panel's opinions between study rounds).^13^

To ensure multiple institutions were represented within our sample, we sorted the list of 55 prospective participants by institution, randomized within each institution, and recruited individuals in batches. During recruitment, prospective participants referred five additional individuals meeting eligibility criteria to us who were subsequently invited to participate.

Prospective participants were invited to participate by email and contacted up to three times. Interested individuals clicked an embedded link to confirm they met our definition of clinician, provide demographic data, and give consent.

### 2.3 Semi‐structured interviews

The interview guide was developed based on a review of the literature on AD biomarker disclosure and tau PET (Supplemental Material). Expert clinicians were asked how they would use tau PET clinically and communicate results to cognitively impaired patients.

Interviews were conducted by CE between October 2023 and July 2024; they were recorded and professionally transcribed. Interviews were conducted until we reached thematic saturation, the point at which conducting additional interviews did not generate new data.

We used deductive thematic analysis^16^ to look for patterns in the data and map data to predefined themes, guided by a published five‐step approach for returning AD biomarker results.^3^ CE created the first draft of the codebook, a taxonomy for categorizing qualitative data,^17^ which defined codes and provided examples. The research team reviewed and revised the codebook until reaching consensus. Using the codebook, two authors (CE, MK) double‐coded a subset of four transcripts and met regularly during this process to compare coding, discuss discrepancies, and refine the codebook to rectify ambiguities, eliminate redundancy, and increase comprehensiveness. Once the codebook was finalized, CE and MK single‐coded the remaining transcripts. We used NVivo version R1/2020 (QSR International) to manage coding.

After coding, the research team reviewed the data and, identifying shared approaches, developed candidate practices (i.e., how to communicate a result) and statements (i.e., language used to explain results) for the return of tau PET results. The statements described several possible results: tau PET results only (detected = T+, not detected = T−) and both amyloid and tau PET results together (A+T+, A−T+, A+T−, or A−T−).

### 2.4 Surveys

The expert clinicians who completed interviews were invited to participate in two survey rounds via Qualtrics.

In Round 1, panelists were given five options for binary terms that could be used to give a qualitative global summary of a tau PET result: elevated/not elevated, positive/negative, abnormal/normal, tau present/not present, or significant/not significant. Additionally, panelists rated the importance of the candidate practices and acceptability of the candidate statements on five‐point Likert‐type scales (1 = low importance/acceptability to 5 = high importance/acceptability); there was a “neutral” option. Panelists were asked to provide comments explaining any ratings less than 4.

We prespecified items receiving ratings of 4 or 5 from at least 80% of panelists would be considered to have achieved consensus. Items receiving ratings of 4 or 5 for less than 50% were considered low support. Mixed‐support items were those with ratings of 4 or 5 for 50% to 79% of responses. Items receiving low support were to be removed (though we made an exception, noted below) and those with mixed support were to be revised in response to participant comments and reconsidered in the next round.

To allow panelists to share knowledge asynchronously, we distributed a summary of Round 1 data prior to Round 2. This included numeric results, a list of items that achieved consensus, a list of items for consideration in Round 2, and de‐identified comments.

In Round 2, panelists rated items on five‐point Likert‐type scales and could comment on all items, regardless of their rating. We used the same prespecified cut‐off for determining consensus and removed items that received low or mixed support.

## 3 RESULTS

Of the 60 prospective participants contacted, three were ineligible because they had not evaluated memory patients within the prior 12 months. Four declined, one was lost to follow‐up, and 32 did not respond to recruitment. Twenty expert clinicians completed the semi‐structured interviews. Interviews lasted 52 min on average (range: 40 to 87 min). Of the 20 interviewed, 18 (16 neurologists, one radiologist, and one geriatric psychiatrist) completed both survey rounds. See Table 1 for additional demographic information.

**TABLE 1:** Sample characteristics of Delphi panelists.

Characteristic	*N* (%)
**Gender**	
Male	15 (83%)
Female	3 (17%)
**Race**	
White	18 (100%)
**Ethnicity**	
Hispanic or Latino	1 (6%)
Not Hispanic or Latino	17 (94%)
**Years as a practicing clinician**	
<10	2 (11%)
10 to 20	7 (39%)
21 to30	4 (22%)
>30	5 (28%)
**Clinical specialty**	
Neurology	16 (88%)
Radiology	1 (6%)
Geriatric psychiatry	1 (6%)
**Experience disclosing Alzheimer's biomarkers**	
Yes	18 (100%)
No	0 (0%)
**Biomarkers disclosed**	
Magnetic resonance imaging	17 (94%)
Amyloid positron emission tomography (PET)	18 (100%)
Tau PET	15 (83%)
Cerebrospinal fluid markers	15 (83%)
Blood‐based markers	10 (56%)
Other	2 (11%)
**Which most closely reflects your definition of Alzheimer's disease?**	
Clinical characteristics: Alzheimer's disease is defined by signs and symptoms	0 (0%)
Biological characteristics: Alzheimer's disease is defined by amyloid and tau	13 (72%)
Both: Alzheimer's disease is defined by both clinical and biological characteristics.	5 (28%)
**“In order to have Alzheimer's disease, a person must have both amyloid and tau.”**	
Strongly agree	11 (60%)
Somewhat agree	3 (17%)
Neither agree nor disagree	1 (6%)
Somewhat disagree	3 (17%)
Strongly disagree	0 (0%)

### 3.1 Interviews

When discussing the clinical utility of tau PET, expert clinicians emphasized regional specificity. Comparing tau PET to amyloid PET, they noted tau PET provided more prognostic information and that the anatomical progression of tau was more related to symptoms.

Many explained that how they communicated a result depended on the patient's level of cognitive impairment: When patients have MCI, tau PET is most useful for prognosis and treatment‐related decision‐making, whereas for patients with dementia, tau PET is more useful for achieving diagnostic certainty.

The expert clinicians described their processes for returning tau PET results. Common elements included providing a global summary or overall impression of the patient's scan, highlighting specific areas of tau accumulation, and explaining what this meant for the patient's diagnosis and prognosis. The expert clinicians provided specific language for communicating results.

### 3.2 Surveys

In Round 1, of the 25 candidate items presented to panelists (Table 2), nine practices and four statements achieved consensus. One item received low support, and 11 received mixed support.

**TABLE 2:** Round 1 candidate practices and statements for communicating tau PET results.

Candidate practices for returning tau PET results	Describe the biological basis of AD. Describe the limitations of tau PET. Give a global or overall summary of the patient's scan. Show the patient their scan. Describe the patient's regional accumulation of tau. Compare the patient's scan with an example scan with and without evidence of tau. Explain how the patient's symptoms are or are not explained by regional accumulation of tau. Provide a quantitative measure of the patient's scan. Explain the meaning of the patient's result for diagnosis. Explain the likelihood that AD is a cause of the patient's symptoms. Explain the meaning of the patient's result for prognosis. Explain how the patient's result informs treatment options.
Candidate statements for communicating a T+ result	(Individual has MCI or dementia): “There was evidence of tau. This result indicates that Alzheimer's disease is a cause of your symptoms.” (Individual has MCI): “Based on what is known about the role of tau in the Alzheimer's disease process, your symptoms are likely to worsen over the next three to five years.”
Candidate statements for communicating a T− result	(Individual has MCI or dementia): “There is no evidence of tau, this means your symptoms are not caused by Alzheimer's disease.”
Candidate statements for communicating an A+T+ result	(Individual has MCI or dementia) “There is evidence of both amyloid and tau. This suggests that Alzheimer's disease is a cause of your symptoms.” (Individual has MCI) “Based on what we know about amyloid and tau in the Alzheimer's disease process, your symptoms are likely to worsen over the next three to five years.”
Candidate statements for communicating an A+T− result	(Individual has MCI): “There is evidence of amyloid, but not tau. This suggests an early stage of Alzheimer's disease.” (Individual has MCI): “It could be a few years before your symptoms progress.” (Individual has MCI): “Your symptoms may be caused by Alzheimer's disease, or they may be caused by another disease.” (Individual has dementia): “Because there is evidence of amyloid, you have some Alzheimer's changes. However, your negative tau scan means it is most likely that some other disease is causing your symptoms.” (Individual has dementia): “If Alzheimer's disease were a cause of your symptoms, we would expect evidence of both amyloid and tau.”
Candidate statements for communicating an A−T+ result	(Individual has MCI or dementia): “There is evidence of tau but not of amyloid. This means Alzheimer's disease is likely not a cause of your symptoms.” (Individual has MCI or dementia): “It's not certain what this result means, but it likely indicates a disease other than Alzheimer's disease is a cause of your symptoms.”
Candidate statements for communicating an A−T− result	(Individual has MCI or dementia): “Neither amyloid nor tau were present. This indicates that Alzheimer's disease is not a cause of your symptoms.”

Abbreviations: AD, Alzheimer's disease; MCI, mild cognitive impairment; PET, positron emission tomography.

We decided to revise rather than remove the low‐support item for reconsideration in Round 2, because it was the only statement for communicating a T− result, and – given the study's objective – we deemed it important to attempt to reach consensus on this point. Round 2 ultimately included 12 revised items and one new item (Table 3). Of these, three practices and four statements achieved consensus.

**TABLE 3:** Round 2 candidate practices and statements for communicating tau PET results.

Candidate practices for returning tau PET results	Amyloid information (PET, CSF, or plasma) is necessary if tau PET information is being returned to a patient Compare the patient's scan with an example scan without evidence of tau.* Providing a quantitative measure of the patient's scan (e.g., SUVR, DVR, Centaurs).* Discuss with the patient how the tau scan may explain their symptoms.*
Candidate statements for communicating a T+ result	(Individual has MCI or dementia): “There was evidence of tau. This result indicates that Alzheimer's disease may be a cause of your symptoms, but further testing is needed to confirm the cause.”* (Individual has MCI): “Based on what is known about the risk associated with a tau PET scan like yours, your symptoms are likely to worsen over the next three to five years.”*
Candidate statements for communicating a T− result	(Individual has MCI or dementia): “There is no evidence of tau, which means your symptoms are unlikely to be caused by Alzheimer's disease.”*
Candidate statements for communicating an A+T+ result	None – Statements achieved consensus in Round 1
Candidate statements for communicating an A+T− result	(Individual has MCI): “There is evidence of amyloid, but not tau. This could suggest early Alzheimer's changes, but you may have another condition that could be causing your symptoms. Further testing could help sort out the causes.”* (Individual has MCI): “There is evidence of amyloid, but not tau. Without more information, it's difficult to predict how, if at all, your symptoms might progress.”* (Individual has dementia): “There is evidence of amyloid, but not tau; we cannot say with certainty what is causing your symptoms. This suggests that Alzheimer's disease might be a cause, but there may be another disease causing or contributing to your symptoms. Further testing could help sort out the causes.”* (Individual has dementia): “If Alzheimer's disease were the main cause of your symptoms, we would usually expect evidence of both amyloid and tau.”*
Candidate statements for communicating an A−T+ result	(Individual has MCI or dementia): “There is evidence of tau but not of amyloid. This is not a typical Alzheimer's disease pattern and we need to gather more information to understand what is causing your symptoms.”* (Individual has MCI or dementia): “This result likely indicates a disease other than Alzheimer's is a cause of your symptoms, though Alzheimer's disease cannot be ruled out.”*
Candidate statements for communicating an A−T− result	None – Statement achieved consensus in Round 1

Abbreviations: CSF, cerebrospinal fluid; DVR, distribution volume ratio; MCI, mild cognitive impairment; PET, positron emission tomography; SUVR, standardized uptake value ratio.

* Item revised based on panelists’ Round 1 comments.

Across both rounds, a total of 12 practices and eight statements for communicating tau PET results to cognitively impaired patients achieved consensus (Table 4).

**TABLE 4:** Consensus‐derived practices and statements for communicating tau PET results.

Practices for returning tau PET results	Amyloid information (PET, CSF, or plasma) is necessary if tau PET information is being returned to a patient Describe the biological basis of Alzheimer's Describe the limitations of tau PET Give a global or overall summary of the patient's scan Show the patient their scan Describe the patient's regional accumulation of tau Compare the patient's scan with an example scan without evidence of tau. Discuss with the patient how the tau scan may explain their symptoms. Explain the meaning of the patient's result for diagnosis Explain the likelihood Alzheimer's is a cause of the patient's symptoms Explain the meaning of the patient's result for prognosis Explain how the patient's result informs treatment options
Statement for communicating a T+ result	(Individual has MCI): “Based on what is known about the risk associated with a tau PET scan like yours, your symptoms are likely to worsen over the next three to five years.”
Statements for communicating a A+T+ result	(Individual has MCI or dementia) “There is evidence of both amyloid and tau. This suggests that Alzheimer's disease is a cause of your symptoms.” (Individual has MCI) “Based on what we know about amyloid and tau in the Alzheimer's disease process, your symptoms are likely to worsen over the next three to five years.”
Statements for communicating a A+T− result	(Individual has MCI): “Your symptoms may be caused by Alzheimer's disease, or they may be caused by another disease.” (Individual has MCI): “There is evidence of amyloid, but not tau. This could suggest early Alzheimer's changes, but you may have another condition that could be causing your symptoms. Further testing could help sort out the causes.” (Individual has dementia): “If Alzheimer's disease were the main cause of your symptoms, we would usually expect evidence of both amyloid and tau.”
Statement for communicating a A−T+ result	(Individual has MCI or dementia): “There is evidence of tau but not of amyloid. This is not a typical Alzheimer's disease pattern and we need to gather more information to understand what is causing your symptoms.”
Statement for communicating a A−T− result	(Individual has MCI or dementia): “Neither amyloid nor tau was present. This indicates that Alzheimer's disease is not a cause of your symptoms.”

Abbreviations: CSF, cerebrospinal fluid; MCI, mild cognitive impairment; PET, positron emission tomography.

### 3.3 Practices when returning tau PET results

Twelve of 16 candidate practices for returning tau PET results achieved consensus. Practices that reached consensus were those that panelists found useful or effective in their own practice. For example, reflecting on why it was important to compare a patient's scan with an exemplar, one panelist commented, “In my experience it is very helpful to show participants examples of ‘elevated’ and ‘non‐elevated’ scans before showing them their own images.” Other panelists (*N* = 6/8 comments) echoed this sentiment about the importance of comparing a scan to an exemplar.

In response to the candidate practice “[d]iscuss how the tau scan may explain the patient's symptoms,” panelists also emphasized the importance of patient‐centered care (*N* = 3/4 comments). One panelist stated, “This is a great opportunity to empower patients and care partners by educating them about the disease, given the strong link between topography of tau and symptoms.” Another emphasized, “THIS is what patients want. “How bad is it? What does that mean?” This is a driving factor behind doing the scan.”

One of the three revised candidate practices included in Round 2 – “[p]roviding a quantitative measure of the patient's scan” – did not achieve consensus and was removed. In the comments, panelists (*N* = 5/13 comments) described its inclusion as premature because “…we currently do not have a good way to do this.” Additionally, panelists (*N* = 7/13 comments) felt the meaning of regional tau accumulation could not be conveyed in a quantitative measure. One explained, “As opposed to amyloid PET, it is the topographic extent of tauopathy that is informative, and that will not be a single number.”

### 3.4 Clinician statements for returning tau PET results

In Round 1, of the five options for binary terms that could be used to give a qualitative global summary of a tau PET result, only elevated/not elevated achieved consensus. Based on panelist feedback suggesting a ternary rather than binary summary would be preferable, in Round 2 we asked whether the terms “elevated/intermediate/not elevated” were preferable to elevated/not elevated. While not achieving consensus, nearly two‐thirds of respondents (61%, *n* = 11) preferred the former to the latter. Three respondents provided their own preferred terminology: “elevated in neocortex/elevated in medial temporal lobe/not elevated.”

Eight of 22 statements for returning tau PET results achieved consensus. Panelists’ feedback was specific to the result being communicated.

- T+: Panelists achieved consensus on one statement describing the likelihood of symptoms worsening within the next 3 to 5 years for individuals with MCI. They did not endorse telling cognitively impaired patients that a T+ result “indicates AD may be a cause of your symptoms, but further testing is needed to confirm the cause.” Panelists (*N* = 9/9 comments) emphasized further testing was likely not needed because (a) “…any discussion of a tau PET would have been preceded by a discussion of an amyloid PET that showed elevated amyloid…” or (b) “…If it is AD pattern tau in a patient with AD pattern deficits, this may be sufficient [for confirming the cause of symptoms].”
- T−: Panelists did not achieve consensus on either of the two candidate statements for communicating a T− result. Panelists (*N* = 8/9 comments) stated that, due to limitations of current tracers, such a result is inconclusive. Interpretation also depended on the patient's level of cognitive impairment. Illustrating this, a panelist commented in Round 2, “Many of the current tau PET tracers are insensitive to Braak III‐IV and earlier tau pathology, which is the level of tau that most corresponds with MCI.” They continued, “If the person has dementia‐level impairment, then this statement would be more acceptable, as these people usually have more advanced tau deposition that can be detected by tau PET.”
- A+T−: Panelists agreed that if a patient with MCI received an A+T− result, AD might be contributing to symptoms, but further testing would be useful to assess whether there were other contributors. They agreed that the implications of this result for prognosis were uncertain (*N* = 5/7 comments). One commented, “With amyloid being elevated, there could still be some risk of progression, though maybe not as high as someone with both amyloid and tau … [I]t would be difficult to predict how the individual's symptoms would progress.” By comparison, if a patient had dementia, panelists agreed it was likely a condition other than AD that was primarily driving symptoms. If AD was the main cause, they would expect evidence of both amyloid and tau.
- A−T + : Panelists observed an A−T+ result occurred infrequently and was not typical of AD. Panelists (*N* = 3/4 comments) stated that discussion of the cause of symptoms would depend on the specific scan findings. One wrote, for instance, that “[h]igh tau with negative amyloid suggests a false negative amyloid. Weak tau with negative amyloid is more typical of other neurodegenerative diseases.” Several (*N* = 5/9 comments) panelists deemed the statement “AD cannot be ruled out” unacceptable, noting, for example, that this was “[t]echnically correct, but practically not helpful. It relegates us back to the ‘an autopsy is required to exclude Alzheimer disease’ days.”
- A + T + : Panelists agreed an A+T+ result indicated AD was the main cause of a patient's symptoms and that for patients with MCI, this result suggests symptoms will worsen over the next 3 to 5 years. These statements achieved consensus in the first round.
- A−T−: Panelists agreed in Round 1 that if a cognitively impaired patient had an A−T− result, AD was not a cause of the patient's symptoms. One panelist suggested more information should be gathered to determine the cause of a patient's cognitive impairment.

## 4 DISCUSSION

We conducted a modified Delphi process, including both interviews and surveys, with expert clinicians to develop novel guidance for communicating tau PET results to cognitively impaired patients that goes beyond the interpretation provided in the FDA‐approved label for flortaucipir (Tauvid) and better reflects current understandings of tau PET results. Twelve practices and eight statements achieved consensus. These findings may inform practice in the clinic and in research studies.

Reflecting areas of consensus in the field more broadly,^18^ panelists felt it was necessary to have amyloid information if tau PET information was being returned and noted the value of regional tau accumulation. The temporal association of tau PET with symptom severity and disease progression was reflected in statements that achieved consensus for the A+T+ compared to the A+T− statements. Experts agreed that an A+T+ biomarker profile would indicate the patient's symptoms would likely worsen in the near future. This is consistent with findings from a large, international study of 6514 participants across the cognitive spectrum, which found a high risk of clinical progression for cognitively impaired and unimpaired participants with tau PET positivity.^7^

On the other hand, for A+T− biomarker profiles, panelists noted the uncertainty about when symptoms might worsen. The results also identified areas of mixed opinion, including the interpretation of discordant amyloid and tau results and its meaning for determining whether AD was the primary cause of a patient's symptoms. Consistently, for items that did not achieve consensus, panelists cited a need for more information to make a statement about the results, highlighting the context‐specific nature of tau PET. A growing body of evidence suggests clinical, genetic, and demographic factors impact prognosis and may relate to tau accumulation. For example, Ossenkoppele et al. characterized the prevalence of tau positivity within 12,048 participants across the cognitive spectrum and found that age and amyloid status had the strongest associations with tau positivity.^19^ This and similar work may be used for refining the consensus statements and practices going forward.

Though the goal of this modified Delphi study was to provide guidance on how to communicate tau PET results to patients – and not to evaluate or develop appropriate use criteria (AUC) – we note that our findings are generally concordant with the Alzheimer's Association and Society for Nuclear Medicine and Molecular Imaging Workgroup (AA‐SNMMI) AUC for tau PET.^18^ The panelists emphasized in interviews that tau PET should not be conducted until a comprehensive clinical examination has been carried out. The AA‐SNMMI AUC similarly note:

“The entirety of these clinical data [detailed medical and neurobehavioral history, physical examination, mental status testing, blood tests to rule out potentially reversible causes of cognitive impairment, and structural brain imaging] are required to optimally integrate amyloid/tau PET results into clinical decision making regarding diagnosis and patient management.”^18^

Similarly, panelists in our study highlighted the utility of tau PET for determining prognosis; ultimately, two statements regarding prognosis for patients with MCI achieved consensus. The AA‐SNMMI AUC includes scenarios, two of which highlight the appropriateness of using tau PET for prognosis. Overall, there were no glaring areas of discordance between the AA‐SNMMI AUC and our findings.

Our results also align with an international, online survey of clinicians and researchers (*n* = 268) conducted in 2023. This survey collected perspectives on the possible future role of tau PET in clinical practice and trials.^20^ Respondents broadly expressed enthusiasm for tau PET. They emphasized its clinical value for disease staging, diagnosis, monitoring, and prognostication for cognitively impaired patients. Respondents felt tau PET would, in addition to its clinical value, likely have an important role in clinical trials, particularly for participant selection and endpoint measurement.^20^ Our results build on these survey findings by providing initial guidance for communicating the important information conveyed by tau PET to cognitively impaired patients.

Our results contrast with FDA‐approved labels for flortaucipir (Tauvid).^5^ Throughout the Delphi process, panelists emphasized the importance of the regional specificity of tau PET and the unique prognostic information it provides. The current FDA‐approved label largely neglects regional specificity, including only neocortical binding (e.g., medial temporal lobe binding is excluded). Further, the FDA‐approved label describes scan results as either “negative” or “positive.” Notably, the negative/positive framing did not achieve consensus among our panelists, whereas elevated/not elevated did. The preference for “elevated/not elevated” may reflect skepticism about the reliability or interpretive clarity of tau PET. Additional exploration into summary language preferences might reveal these reasons. Revisions to FDA‐approved labels may be warranted to more closely align with practice.

Despite consensus for the elevated/not elevated framing during the first round, panelists suggested ternary summary language might be preferred. We followed up in the second round by asking whether the terms “elevated/intermediate/not elevated” were preferable to “elevated/not elevated.” A majority (61%) preferred this ternary language; however, three panelists (17%) offered region‐specific descriptors as their preferred result summary language. This lack of agreement suggests ongoing uncertainty about the interpretation of tau PET, making it difficult to agree on what to communicate.

The guidance presented herein assumes the clinician returning results has access to a clear tau PET visual read. We recognize that, while there is growing consensus tau PET would benefit the care of people with cognitive impairment,^7^, ^18^, ^20^ key barriers to widespread implementation of tau PET exist.^21^, ^22^ First, tau PET is resource intensive, requiring expensive equipment, access to tracers, and trained specialists qualified to conduct tau PET visual reads. Second, available tracers do not detect early tau accumulation^23^, ^24^, ^25^ (Braak stages I–IV^26^), introducing ambiguity about the meaning of T− results.^27^ In the future, should tracers become available that reliably detect earlier Braak stages, clinicians would likely include staging information in their communication of tau PET results. Third, as noted above, there is not currently a harmonized quantitative scaling approach, though efforts are under way.^28^ Though the topographic extent of tau PET is important, the development of clinically meaningful thresholds for quantitative results is also needed. Thresholds could support the use of ternary summary results language (e.g., elevated/intermediate/not elevated). More data are required to develop these cut‐off points and may be drawn from clinical trial data or patient data from those on anti‐amyloid therapies.

## 5 LIMITATIONS

This study has several limitations. First, interviews were conducted with a small number of expert clinicians. Some perspectives may have been excluded based on how we defined expert clinicians (e.g., people who are working in this area but have not published on the topic). Second, the requirement of being a US‐based clinician led to the exclusion of international experts, possibly limiting the perspectives reported.

Third, because the panel included only experts in tau PET and a majority of panelists were neurologists, the feasibility of broadly implementing the consensus‐derived recommendations may be limited to research and specialty‐care settings, as clinicians with less expertise or experience with tau PET imaging may not be adequately prepared or may lack the resources to implement them. Nevertheless, these practices and statements are an important first step. As tau PET is more broadly adopted, future research should aim to adapt and build on these findings for the primary care setting, as primary care clinicians are often first to hear of cognitive complaints.

Fourth, clinicians were asked how they would communicate tau PET results to hypothetical patients. While they were able to do this in general terms, they noted that additional clinically relevant details of a patient's case could provide important context to result disclosure. Finally, this study did not include patient or caregiver perspectives. Future work should include patients and caregivers to ensure the return of results meets their needs. Studies of return of tau PET results, as occurred for amyloid PET, are recommended to understand the patient and care partner experience.

Tau PET offers unique and important diagnostic and prognostic information, but guidance for how to communicate such results to cognitively impaired patients is lacking. Our results begin to fill this gap by identifying consensus‐based practices and statements selected by clinicians with expertise in human tau imaging. As new tracers and treatments become available, this guidance may evolve, or new guidance may be needed.

## CONFLICT OF INTEREST STATEMENT

The authors declare no conflicts of interest. Author disclosures are available in the Supporting Information.

## CONSENT STATEMENT

All human subjects provided informed consent.

## Supporting information



Interview Guide for Tau Modified Delphi

Supporting Information
