# Supplementary material for: Expert consensus on communicating tau PET results to persons living with MCI or dementia: Findings from a modified Delphi study
Source: Alzheimers Dement. 2026 Apr 8;22(4):e71321. doi: 10.1002/alz.71321 (PMC13060757; doi:10.1002/alz.71321)
Supplement: Supplementary file 1 — Interview Guide for Tau Modified Delphi [file ALZ-22-e71321-s001.docx]

**Interview Guide for Tau Modified Delphi**

Thank you for taking the time to talk with me. The goal of this study is to develop expert guidance on prescribing tau PET imaging and returning the results to individuals. To achieve this, we are learning experts’ perspectives on prescribing tau PET and returning the results. Your expertise in human tau PET imaging and clinical experience are why you’re being interviewed for this study.

As a reminder, I’ll be recording this interview. I expect it will last about an hour. You’re free to stop the discussion at any time. You don’t have to answer any questions if you’d prefer not to. When we transcribe the interview, we will remove any personal or patient identifying information.

Before we start, I also want to let you know that I am not a clinician. Some of the questions I’ll ask may have obvious answers, but I’d like to ask them anyway to make sure I get the full picture of your experience, and I don’t want to make any assumptions. I also want to emphasize there are no right or wrong answers. We expect a range of responses. Our goal is descriptive and not evaluative.

For the purposes of this interview, I want you to imagine that there *aren’t* institutional or insurance barriers to prescribing tau PET imaging. This interview consists of 3 parts. In the first, I’ll ask you about clinical uses of tau PET in patients with cognitive impairment. Next, I’ll ask you about uses of tau PET in cognitively unimpaired people. Last, I’ll ask about the future of tau PET. Does that sound good? Is it alright if I start recording now?

OK, let’s get started.

**Prior Experiencing Disclosing Tau PET**

- Have you returned tau PET results previously?
  - *If yes*: About how many tau scan results have you returned?
  - *If yes*: Who have you returned tau scan results to?
    - E.g., Cognitive status (dementia, MCI, cognitively unimpaired), Setting (research, clinical…)

**Clinical Uses of Tau PET: Cognitively Impaired Patients**

Given your knowledge of tau imaging in humans, I want to ask you about how you might use a tau PET scan in different settings. In this set of questions, imagine you’re seeing a patient who has cognitive impairment and who you suspect, based on signs and symptoms, has Alzheimer’s disease. The patient says to you that they want to know the cause of their cognitive impairment.

Assume that amyloid and tau PET imaging are readily available and reimbursed by insurance, and patients are open to learning the results. The decision I’d like to focus on is whether to order a tau PET scan.

What characteristics of a patient with cognitive impairment would influence whether you would order a tau PET scan?

*Use below as probes if needed (make sure interviewee addresses all below)*

- Clinical presentation (phenotype e.g., amnestic, aphasic, dysexecutive, etc.)
- Stage or functional status? (e.g., MCI, dementia)
- Age of patient?
- Results of other tests? (e.g., neuropsychological testing, blood tests, FDG-PET, MRI, *APOE* status)
- Family history?
- Other?

Does the availability of pharmacological treatment for Alzheimer’s disease influence your decision to order a tau PET scan?

- How so?

Again, imagine that there aren’t institutional or insurance barriers to tau PET imaging. Let’s say you decide to order a tau scan in the work up of a patient with cognitive impairment that you suspect, based on signs and symptoms, has Alzheimer’s disease.

At what point in the evaluation process would you order the tau scan?

- How would you use the tau scan results in your diagnostic process?
- What other information (including additional biomarker tests) would you want to make your diagnosis or would be important as part of the patient evaluation process?

*Follow up on if different between MCI and dementia*

What clinical value does a tau scan provide?

- (Could be predictive value for progression, validating specific symptoms, etc.)

Let’s assume you ordered a tau PET scan, got the results and now are going to discuss the results with the patient. I want to ask about your general approach to communicating the results.

- First, what terms would you use to describe results?
  - Positive/negative, elevated/not elevated, something else?
  - How would incorporate prognosis into conversation? How would describe prognosis to patient?
- What would be your main goals for this conversation?
  - Are there any key concepts you would emphasize and include in all discussions?
- If the scan showed evidence of tau, what would you say?
  - *If disclosed tau previously:* Do you have any particular words or phrases you use?
  - Would you…
    - *If didn’t address earlier above:* Go beyond a binary outcome (positive/negative), such as using Braak staging or describe regional tau deposition?
      - Why?
    - Consider discussing any form of quantification of the scan result (e.g., centiloids, SUVR, etc.)?
      - Why?
    - Show them an image of their tau scan?
      - Why?
    - Compare their tau scan to a typical scan with or without evidence of tau?
      - Why?
- If the scan did not show evidence of tau, what would you say?
  - *If disclosed tau previously:* Do you have any particular words or phrases you use?
  - Would you…
    - *If didn’t address earlier above:* Go beyond a binary outcome (positive/negative), such as using Braak staging or describe regional tau deposition?
      - Why?
    - Consider discussing any form of quantification of the scan result?
      - Why?
    - Show them an image of their tau scan?
      - Why?
    - Compare their tau scan to a typical scan with or without evidence of tau?
      - Why?
- How would your approach to telling a patient their tau result depend on other clinical features…

*Use below as probes if needed (make sure interviewee addresses all below)*

- - Clinical presentation (phenotype e.g., amnestic, aphasic, dysexecutive, etc.)
  - Clinical stage or functional status (e.g., MCI, dementia)
  - Age of patient
  - Results of other testing (e.g., neuropsychological testing, blood tests, FDG-PET, MRI, *APOE* status)
  - Family history
  - Perceived demeanor of the patient and caregiver (e.g., are they anxious?)
  - Other?

Imagine the patient has had both amyloid and tau testing?

- What would you tell them if the results showed both amyloid and tau are present?
- What would you tell them if the results showed neither amyloid nor tau were present?
- What would you tell them if amyloid was present and tau was not?
- How about it tau was present and amyloid was not?

After returning the result, what do you anticipate the patient or someone accompanying them might ask you?

- *If disclosed tau previously:* When you’ve disclosed tau results previously, what questions frequently come up?

We’ve so far been talking about using tau PET for patients with MCI and dementia. I’d like to switch now and ask about the role of the scan in someone without cognitive impairment.

- Are there clinical scenarios in which you’d order a tau PET for a cognitively unimpaired patient?
  - *Ask them to explain answer*

*Use below probes if necessary (make sure interviewee addresses all below)*

- - Cognitive complaints/Subject cognitive decline
  - Age of patient
  - Results of other testing (e.g., neuropsychological testing, blood tests, FDG-PET, MRI, *APOE* status)
  - Family history
  - Other?

**Uses of Tau PET: Cognitively Unimpaired Research Participants**

Now, I want you think about a *cognitively unimpaired* adult who is in the process of enrolling in a research study. The eligibility criteria include a tau scan result suggesting at least Braak III/IV.

Participants will undergo tau PET and learn the scan results, that is whether they have adequate tau to participate in the study. Assume that disclosure will only go forward if the person is otherwise appropriate (for example, has passed psychological screening), and the study has access to ideal tracers (even if they don’t exist today).

- What information do you think is needed to educate a *cognitively unimpaired* person prior to giving consent to learn their tau results?

Imagine the prospective participant only had tau imaging done as part of the screening process and has chosen to learn the result. They have not undergone any amyloid testing.

- What, if any, information about the participant would you want to know before disclosing?
  - E.g., family history of dementia, caregiving experience, etc.
- What would you say to them if the scan showed evidence of at least Braak III/IV?
  - Would you say anything different if the participant had subjective cognitive decline and the pattern of tau accumulation aligned with the participant’s reported symptoms?
  - Would you say anything different if the participant had subjective cognitive decline and the pattern of tau accumulation did not correlate with the participant’s reported symptoms?
  - Would you say anything about FDA-approved treatments for MCI and mild dementia? (Assume this wouldn’t make them ineligible for the study.)
- What would you say to them if the scan did not show evidence of at least Braak III/IV?
  - Would you say anything different if the participant had subjective cognitive decline?
  - Would you say anything about FDA-approved treatments?

Now imagine the prospective participant has undergone both amyloid and tau imaging as part of the screening process and has chosen to learn both results.

- What would you say to them if the results showed both amyloid and tau are present, that is unambiguous amyloid binding in the cortex (visual rating of at least 3) and at least Braak III/IV?
  - Would you say anything differently if the participant had subjective cognitive decline?
- What would you say to them if the results showed neither amyloid nor tau were present?
- Let’s say the participant shows evidence of amyloid but not tau. What would you say?
- How about if the participant’s scans show evidence of tau, but not amyloid?

**Challenges in Tau Disclosure + Future of Clinical Disclosure**

I now want to ask about current challenges in the use of tau PET and tau PET disclosure more broadly and get your perspective on what the future may look like.

- How, if at all, does the decision to order tau PET differ from the decision to order amyloid PET? (E.g., for whom…)
- How, if at all, does the type of information needed for consent and pre-test education for tau PET differ from that for amyloid PET?
  - Why?
- Before, we talked about tau testing and disclosure of results in an idealized setting (*test is available, reimbursed, etc. only decision is if you want to order the test or not*). Thinking about things as they currently are, what barriers to the uptake of tau PET testing exist in clinical settings? I’m interested in testing for individuals across the cognitive spectrum.
  - What do you think is needed to meet address these challenges?
    - (Could be what we need to know about the meaning of biomarkers for prognosis, availability of disease-modifying treatments, access for testing, training clinicians, etc.)
- The FDA has approved the tau tracer Tauvid, flortaucipir F18 – the text on the indication reads –“TAUVID is a radioactive diagnostic agent indicated for positron emission tomography (PET) imaging of the brain to estimate the density and distribution of aggregated tau neurofibrillary tangles (NFTs) in adult patients with cognitive impairment who are being evaluated for Alzheimer’s disease (AD)”– In light of what we’ve been talking about, I’d like to ask you to reflect on this indication. Does it reflect how you would use the scan? How you would want to use the scan?
- I’d like to ask you about the Alzheimer’s Association and Society for Nuclear Medicine and Molecular Imaging working group’s proposed Appropriate Use Criteria for tau PET. It says “tau PET should be considered in patients who: (1) have undergone a comprehensive assessment by a dementia expert; (2) Alzheimer’s disease (AD) is a diagnostic possibility but uncertainty remains; and (3) knowledge of tau PET results is expected to help establish diagnosis and guide patient management…Tau PET was considered ‘Appropriate’ to clarify diagnosis in patients with MCI/dementia under age 65 or those with atypical presentations; and to inform prognosis in MCI or dementia due to suspected AD.” In light of what we’ve been discussing, I’d like you to reflect on these proposed criteria. Does it reflect how you would use the scan? How you would want to use the scan?
- There have and continue to be advancements in blood-based biomarkers. How do you envision their use in clinical care?
  - - How might this impact the use of tau PET?

**Wrap Up**

- How do you think the availability of anti-amyloid therapies will impact what we’ve talked about today?
- Is there anything we haven’t covered that’s important to you about these topics that you’d like to talk about?
- Is there anything else you’d like to share with me?

Thank you for talking with me today.

We are still in the process of interviewing experts about their perspectives regarding tau PET disclosure. We will review the data from these interviews to identify common themes or ideas and then summarize these findings into elements to be included in Tau PET imaging disclosure materials for patients and participants. We will then reach back out to you to get your feedback on these elements. We’ll be conducting the next steps of this study through surveys we’ll send you via email, so be on the lookout for communication from us. We also need a little more information to compensate you for this interview and the upcoming surveys. My study coordinator will be in contact to get this from you.

Thanks again for your participation and taking the time to talk with me.
